# Longitudinal trends using a point-of-care gelatin-based model for ultrasound-guided central venous catheter insertion

**DOI:** 10.1080/10872981.2021.1924350

**Published:** 2021-05-07

**Authors:** Richard P. Ramonell, Matthew Schimmel, Meredith Greer, Caroline G. Coleman, William S. Bender, Lisa M. Daniels

**Affiliations:** a Division of Pulmonary, Allergy, Critical Care and Sleep Medicine, Emory University, Atlanta, GA, USA; b Department of Medicine, Emory University, Atlanta, GA, USA

**Keywords:** Simulation training, medical education, gelatin, central venous catheters, point-of-care ultrasound, internal medicine

## Abstract

Ultrasound (US)-guided central venous catheter (CVC) insertion is a procedure that carries the risk of significant complications. Simulation provides a safe learning atmosphere, but most CVC simulators are not available outside of simulation centers. To explore longitudinal trends in US-guided CVC insertion competency in internal medicine (IM) interns, we studied the use of a low-fidelity, gelatin-based, US-guided CVC insertion simulation model combined with a simulation curriculum. This prospective observational study of IM interns was performed over the course of one academic year. Interns (n = 56) underwent model-based, US-guided procedure simulation training program and a repeated training course prior to their intensive care unit (ICU) rotation. CVC insertion competency at different timepoints was recorded. Survey data about intern experience and attitudes were also collected. Out of the 56 interns initially trained, 40 were included in the final analysis. Across all outcomes, interns experienced skill atrophy between initial training and the beginning of their ICU month. However, by the end of the month, there was a significant improvement in competency as compared to initial procedural training, which then waned by the end of the intern year. Attitudes toward the model were generally positive and self-reported confidence improved throughout the course of the year and correlated with objective measures of competency. Over the course of their intern year, which included simulation training using a gelatin-based model, interns demonstrated consistent competency trends. The use of a gelatin-based CVC insertion simulation model warrants further study as an adjunctive aid to existing simulation training.

## Introduction

Ultrasound (US)-guided central venous catheter (CVC) insertion is a common inpatient procedure that carries the risk of significant complications, including infection, arterial puncture, thrombosis, bleeding, and pneumothorax. The strongest predictor for these complications is the number of unsuccessful insertion attempts, a number that directly correlates with the provider’s procedural experience[1].

The American Board of Internal Medicine requires the competency of US-guided CVC insertion during internal medicine (IM) residency training and encourages residency programs to teach trainees through initial simulation followed by supervised active participation[2]. Simulation provides a safe learning environment where learners can practice skills, develop new techniques, and receive feedback on their performance in a low stakes environment. While several studies have shown simulation in medical education to be an effective method in gaining procedural competency for US-guided CVC insertion, IM residents’ opportunities for simulation are limited by both time and resources [3–11].

There are multiple competing factors limiting the IM resident simulation time. First, IM residents have significant educational and clinical responsibilities, resulting in less time available for procedural simulation [12,13]. In addition, simulation centers are experiencing increased numbers of learners with increased simulation needs, resulting in less simulation center time available for each individual IM resident[12]. Furthermore, simulation centers are often isolated from clinical sites, constraining IM residents’ opportunities for simulation to non-clinical time. Given these time constraints, US-guided CVC insertion simulation is often limited to a single training session at the beginning of the academic year.

Following this single training session, IM residents may participate in supervised active US-guided CVC insertion at variable times throughout the academic year. However, during the time between simulation training and active supervised participation, residents’ procedural skills decline to the point that they may lose all competency gained from simulation within months [14,15]. Therefore, for residents that do not participate in supervised active US-guided CVC insertion at regular intervals, simulation ‘boot camps’ that occur only once at the beginning of the year are of little benefit to the learner. In this way, time not only limits the quantity but also the quality of IM residents’ simulation opportunities.

Simulation opportunities are also affected by the availability of resources. There are currently several existing models for US-guided CVC insertion simulation. High-fidelity models, such as cadavers or ccommercially availablemannequins or task trainers, closely mimic human anatomy and require no preparation but are limited by high cost ($1,163 to 8,988 USD) and low portability [16–19]. Low-fidelity models, such as gelatin- or animal tissue-based models, are less anatomically accurate and require preparation but have low cost and high portability. Despite these limitations, when compared to high-fidelity models, low-fidelity models have demonstrated comparable, if not superior, quality [20,21].

Gelatin-based low-fidelity simulation models are not only low-cost and portable, but also have the added benefits of durability and bacterial resistance when compared to animal tissue-based models. In addition, gelatin-based simulation models can also be easily modified to provide the learner with models that reflect inter-patient anatomic variation, providing additional procedural experience[21]. As a result, low-fidelity gelatin-based simulation models are able to overcome multiple time and resource limitations by providing a variety of high-quality models at the point of care.

Little data exist describing the benefits of implementing a low-fidelity gelatin-based model into ICU curricula to help improve US-guided CVC competency. Therefore, medical educators making evidence-based decisions about the simulation training may forego potentially helpful training tools for other strategies. In this prospective observational study, we detail the longitudinal trends in procedural competency, intern confidence, and utility of a low-fidelity, gelatin-based, point-of-care simulation model paired with a longitudinal US-guided CVC insertion simulation training curriculum over the course of one academic year.

## Materials and methods

### Study design

**Figure 1.**
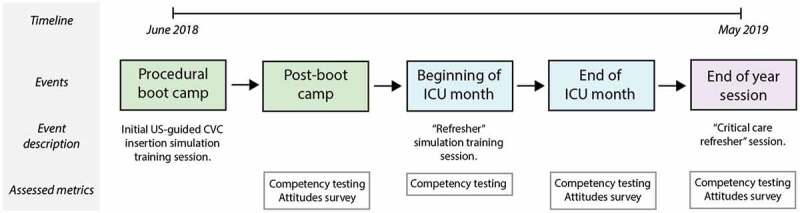
Study design and timeline

We conducted a prospective, single group observational study at a single tertiary care public teaching hospital (Grady Memorial Hospital, Atlanta, Georgia) to study outcomes after incorporating a low-fidelity, gelatin-based, point of care simulation model into an US-guided CVC insertion curriculum among IM interns in their first year of a 3-year training program rotating through a single medical intensive care unit (ICU). Notably, we chose not to use a crossover design nor include a control group as this was deemed by the investigators to be unethical. Multiple well-designed, randomized studies from both medical and surgical education literature have demonstrated the benefits of simulation. We, therefore, chose to use a single experimental group as we believed this would provide useful information to inform future studies without compromising the education of our residents [22–25]. All research was submitted to the Emory Institutional Review Board and was considered exempt from IRB review. The overall study design is displayed in Figure 1.

Prior to starting their residency, interns participated in a boot camp procedure training day at the simulation center. At the conclusion of the session, interns were asked to complete an online survey about their experience and attitudes toward the procedure and gelatin-based model and were tested for procedural competency. Survey data was collected at the conclusion of the interns’ ICU month and at the end of the academic year. Procedural competency was repeated prior to the interns’ ICU month, at the conclusion of their ICU month, and at the end of the academic year.

Following the design of the prototype gel model, a group of Critical Care providers were chosen at random at an academic teaching hospital (Emory Saint Joseph’s Hospital, Atlanta, GA) through which IM trainees do not rotate. These providers were used as a validation cohort to help ensure the validity of the model. After being provided with the model and sufficient time to assess the model to her/his liking, each provider filled out a survey (Supplementary Figure 2) that asked the subject to rate appearance, feel, and utility for teaching specific outcomes on a scale from 1 (inferior to other vascular models) to 3 (superior). Survey responses were positive (Supplementary Table 1) and the model design was finalized.

Prior to starting their residency, interns participated in a ‘boot camp’ procedure training day at the simulation center. During this time, all interns underwent an US-guided CVC insertion simulation training session that included uniform instruction and provided an opportunity to practice using both commercially available mannequins and gelatin-based models. At the conclusion of the session, interns were asked to complete an online survey about their experience and attitudes toward the procedure and gelatin-based model. US-guided CVC insertion competency was also measured at this time using gelatin-based models. Information about boot camp design is included in Supplementary Figure 1.

During the academic year, all interns rotated once through the tertiary care teaching hospital medical ICU, located at a campus separate from the simulation center. At the beginning of this rotation, interns were given a brief US-guided CVC insertion ‘refresher’ simulation session, were shown the location of gelatin-based models in the ICU team room, were encouraged to practice once weekly and immediately prior to any CVC insertion procedure, and repeated the competency testing. At the conclusion of their ICU rotation, interns were again asked to complete an online survey about their experience and attitudes toward US-guided CVC insertion and the model, and procedural competency was tested for the third time using gelatin-based models.

Finally, at the end of the academic year, interns participated in a standardized ‘critical care refresher’ session in the simulation center (Supplementary Figure 1), which included gelatin-based model training. At the conclusion of the session, interns were asked to complete a final online summative survey about their experience and attitudes toward US-guided CVC insertion and the model, and procedural competency was tested using gelatin-based models for the fourth and final time.

### Gelatin-based model construction

**Figure 2.**
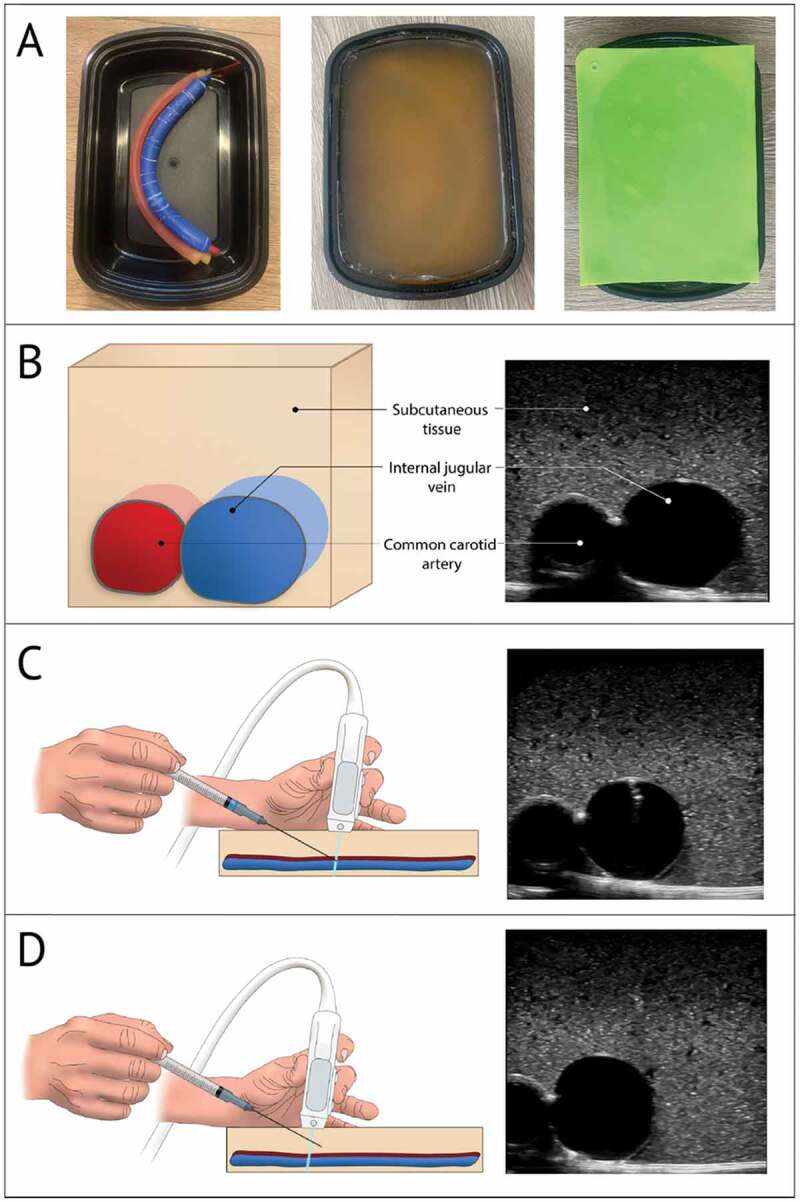
Images of the gelatin-based model

The gelatin-based model design was adapted from a protocol as previously described (Figure 2) [26–31].

(a) Photographs showing, from left to right, the food storage container with balloon and tube in place before the addition of gelatin, the completed gelatin-based simulation model with silicone placemat removed, and completed model with silicone placemat on top. (b) Labeled depiction of gelatin-based model, with illustration on left and US image on right. (c) Illustration of US probe in a transverse position while needle is aspirating simulated venous blood, with accompanying US image. (d) Illustration of US probe in a transverse position with example of incorrect needle tracking, with accompanying US image.

First, the vessels were constructed. The vein was simulated by filling a 25 × 2 cm balloon (Toysmith, Sumner, WA) with 35 mL of water colored blue with food coloring and tying the end closed. The artery was simulated using rubber tubing with a 0.95 cm outer diameter and a 0.64 cm inner diameter cut to a length of 30.5 cm. The tube was filled with water colored red with food coloring and was tied closed at both ends. The simulated vessels were then adhered together in an anatomically correct configuration (vein superficial and lateral to artery) using cyanoacrylate glue (Super Glue Corporation, Ontario, Canada) and were positioned in a 22.4 × 15.5 × 7.1 cm food storage container (Enther, New York, NY).

Next, the gelatin mixture was prepared. In order to create a gelatin-based model that is stable at room temperature while retaining compressibility, appropriate echogenicity on ultrasound, and bacterial resistance, a mixture of 25–35% unflavored gelatin (Knox, Camden, NJ), 2–3% agar (Nature et Plantes, Saint-Paul-lès-Dax, France), and 3–4% sugar-free psyllium hydrophilic mucilloid fiber (Procter and Gamble, Cincinnati, OH) was combined in water. The mixture was placed on low-medium heat, avoiding simmering and boiling, and was stirred until fully dissolved. The mixture was then allowed to cool until the temperature reached < 50°C but the gelatin was still in liquid phase. Finally, 0.1 to 0.5% preservative (Germ-X, Saint Louis, MO) was stirred into the mixture which was then poured into the food storage container until full. The food storage container, gelatin mixture, and submerged vessels were then cooled in the refrigerator at 4°C for 2 hours. Once solidified, a 0.08 cm silicone placemat (TrendBox, USA) was placed on top of the model to mimic the resistance of skin. In total, the materials used to construct each model cost about 6 USD and required 3 hours to create. Four gelatin-based models were available monthly at the start of each clinical rotation.

### Survey design

Survey questions were generated *a priori* and, in some cases, were adapted from attitude questions available in the medical education simulation literature [8,24]. Survey questions focused on the intern’s perceived utility of CVC insertion simulation, subjective recall of number of procedures performed prior to intern year and during their ICU month, confidence in US-guided vascular access procedural competency (including peripheral IV and CVC), and perceived utility of the gelatin-based models. Surveys were composed by the investigators and were reviewed by an expert in medical education for content validity; the surveys were revised based on this feedback and were pretested on two fellows in Pulmonary and Critical Care Medicine prior to distribution. Once finalized, surveys and response data were uploaded to, distributed through, and stored in REDCap (Research Electronic Data Capture).

### Competency testing

Intern CVC insertion competency was assessed at four timepoints: following procedural boot camp, prior to their ICU month, at the end of their ICU month, and at the end of the year (Figure 1). Testing was performed using the gelatin-based model to measure four separate competency metrics: time required to get return of venous blood in the syringe (‘time to flash’), number of needle sticks before flash, accurate identification of the artery and vein based on appearance on US, and adequacy of needle tracking. Five investigators underwent standardized training in administration of the gelatin-based model competency examination and were instructed to use a script prior to testing. Video recordings of the simulated procedure were obtained and were used to resolve any discrepancies during testing. If this occurred, a second investigator was consulted as an adjudicator to determine testing results. All intern videos were stored in a secure online repository.

### Statistical analysis

All statistical analyses were performed using Prism 8 (version 8.2.1, GraphPad). Descriptive statistics of demographic characteristics and venous access competency data were calculated using medians and interquartile ranges (IQR). Data for the number of sticks were non-parametric as measured by Anderson-Darling, D’Agostino & Pearson, and Shapiro-Wilk testing. However, since the median and IQR were the same for several timepoints, 95% confidence intervals (CI) were also included to more descriptively display the data to the reader. Continuous and discrete data from CVC insertion competency testing were compared using Wilcoxon matched-pairs signed rank tests. In all cases, p values < 0.05 were considered statistically significant.

## Results

Between June 2018 and May 2019, 56 categorical, medicine-psychiatry, primary care track, and research track IM interns were enrolled in the study and underwent a standardized, hands-on, US-guided CVC insertion simulation training program. Subject demographic information is included in Table 1. Of the original 56 interns enrolled, 52 (92.9%) completed the post-boot camp survey, 50 (89.3%) completed the post-ICU survey, and 48 (85.7%) completed the end-of-year survey. CVC competency data were available at all four time points for 40 (71.4%) interns and was included in the final analysis.

**Table 1.** Baseline demographic characteristics of interns*

Characteristic	All residents (n = 56)	Completed all CVC testing (n = 40)
Gender – no. (%)		
Male	27 (48.2)	20 (50.0)
Medical school type – no. (%)		
U.S. medical school graduate	53 (94.6)	37 (92.5)
Residency type – no. (%)		
Categorical	44 (78.6)	32 (80.0)
Medicine/psychiatry	2 (3.6)	1 (2.5)
Primary care track	8 (14.2)	5 (12.5)
Research track	2 (3.6)	2 (5.0)
Median number of US-guided procedures completed prior to residency (IQR)* Peripheral IV	1 (0–1)	
Central venous catheter	1 (0–1)	

* Percentages may not total 100 because of rounding.

** Data for baseline number of US-guided procedure completed prior to start of residency were missing for four subjects.

### Central venous catheter insertion competency

**Figure 3.**
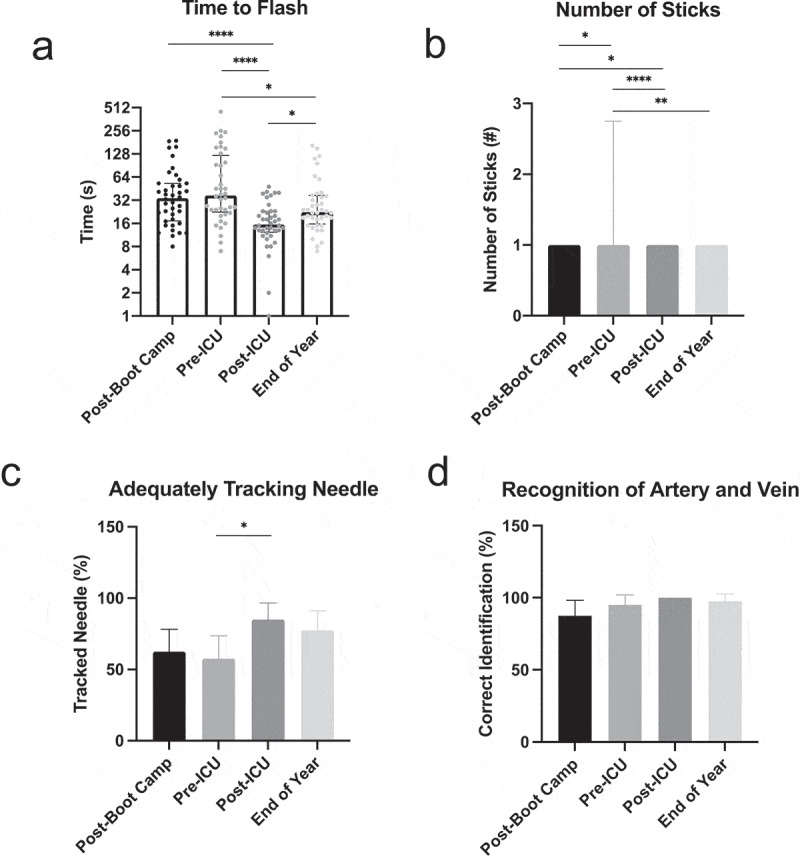
US-guided CVC insertion competency data. Intern (n = 40) CVC insertion competency as measured by median time between the start of the procedure and successful vessel cannulation (a), median number of needle sticks prior to successful venous access (b), percentage of interns using appropriate needle tracking with ultrasound (c), and percentage of interns accurately recognizing vessels using ultrasound (d) at four time points. Error bars indicate IQR in Figure 3a and 3b and 95% confidence interval in Figures 3 C and 3D. * = p < 0.05, ** = p < 0.01, *** = p < 0.001, **** = p. < 0.0001

Overall, the 40 interns that completed US-guided CVC insertion competency testing at all four timepoints improved in their skills from boot camp until the end of year assessment (Figure 3 and Supplementary Table 2). Initially, following procedural training at boot camp, the median time that interns took to access the vein using the gelatin-based model was 34 seconds (Figure 3(a), IQR 17.25–53). Between boot camp and the beginning of their ICU rotation, interns lost skill and took a median time of 37 seconds (IQR 22.5–122.3) to complete the same task, although this was not statistically significant (p = 0.39). By the end of their ICU month, interns showed the greatest improvement in time to flash (median time 15.5 seconds, IQR 12.25–23), which was statistically significant when compared with post-boot camp (p < 0.0001) and the beginning of their ICU month (p < 0.0001) time points. Between the end of their ICU month and the end of the year, interns experienced statistically significant skill atrophy, with a median time to flash of 22.5 seconds (IQR 15.75–36.75, p = 0.02) but did not return to their baseline time to flash from the beginning of the year.

In a similar fashion, interns performed poorly in the number of sticks required to achieve venous access at boot camp (median 1, IQR 1–1, 95% CI 1.1–1.5, Figure 3(b)) which worsened by the beginning of their ICU month (median 1, IQR 1–2.75, 95% CI 1.4–2.5, p = 0.02). Significant improvement in the number of needle sticks was noted at the end of the ICU month (median 1, IQR 1–1, 95% CI 1–1.1) when compared to boot camp (p = 0.047) and the beginning of the ICU month (p < 0.0001). Once again, interns experienced skill atrophy between the conclusion of their ICU month and the end of the year (median 1, IQR 1–1, 95% CI 1–1.3) but remained more proficient when compared to the first recorded timepoint. Although the medians for all timepoints were the same, the statistical differences noted are a result of data skewed towards higher numbers of sticks at earlier timepoints.

Interns also improved their ability to track their needles using the US between the beginning of their ICU month (Figures 3(c), 57.5% correctly tracking needle) and the end of their ICU month (85% correctly tracking needle, p = 0.03) but some skill was lost when the interns were retested at the end of the year (77.5% correctly tracking needle). Finally, interns demonstrated high initial competency when asked to distinguish between the artery and vein using US (87.5% at boot camp) which increased to 100% accuracy at the post-ICU timepoint, although this was not statistically significant (Figure 3(d), p = 0.06). Once competency was achieved in this skill, there was little variance around each timepoint, which is likely related to the ease of acquisition and the reproducibility of this skill in the gelatin-based model.

### Survey responses

**Figure 4.**
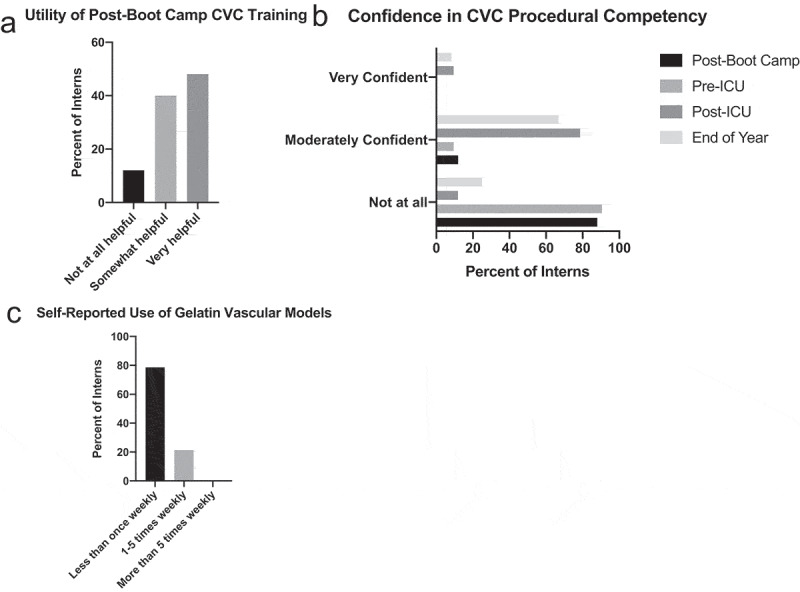
Survey responses. Interns (n = 52) were surveyed at the conclusion of their US-guided CVC insertion ‘boot camp’ training session about perceived utility of the initial training (a). In addition, interns were surveyed about subjective confidence in CVC procedural competency at three time points (c): at the end of the boot camp training session, at the end of their ICU month (n = 50), and at the end of their intern year (n = 48). Interns were also surveyed about self-reported use of the gelatin vascular models at the conclusion of their ICU month (D)

Out of the 52 interns that responded to the post-boot camp survey, most (88%) found the US-guided CVC insertion training ‘somewhat helpful’ or ‘very helpful’ (Figure 4(a)). Prior to the start of their intern year, interns were not very experienced with US-guided procedures and had only attempted an average of only 0.62 US-guided peripheral IV insertions and 0.77 US-guided CVC insertions (Table 1). Accordingly, 88% of the interns were ‘not at all’ confident and 12% were ‘moderately confident’ in their ability to perform US-guided CVC insertion, even after the boot camp simulation session (Figure 4(b)). Interns surveyed were slightly more confident in their ability to perform US-guided IV insertion, with 4% reporting feeling ‘very confident,’ 50% reporting feeling ‘moderately confident,’ and the remainder ‘not at all’ confident in their ability to complete this task.

Following their ICU month, survey respondents (n = 50) did note an improvement in perceived procedural competency with 78.6% of interns reporting feeling ‘moderately confident’ and 9.5% feeling ‘very confident’ in their ability to perform CVC insertion (Figure 4(b)). While the magnitude of the effect of the gelatin-based model on this outcome is impossible to quantify without a control group, 83.3% of the interns did find the models either ‘somewhat helpful’ or ‘very helpful.’ However, in spite of the perceived utility of the models, only 21.4% of the interns used the models at least 1 to 5 times weekly and none of the interns used the model more than 5 times weekly; all remaining interns (78.6%) used the model less than once weekly (Figure 4(c)).

Because of the discrepancy in perceived utility and model use, *post hoc* questions were added to the end-of-year survey to clarify barriers to the use of the gelatin-based model. Of the 48 interns that responded to the end-of-year survey, almost half (47.9%) wanted more ultrasound practice in general during their ICU month. Only a minority of respondents (14.6%) thought access to the model during the ICU month was a barrier to use, and most interns did not want a higher fidelity model (85.4%) nor more supervision for practice (91.7%). 33.3% said that more time to practice during their ICU month would have increased use.

Finally, concordant with the loss of objective competency between the conclusion of their ICU month and the end of the year, interns were less confident in their ability to perform CVC insertion on their end-of-year survey, with 66.7% feeling ‘moderately confident’ and only 8.3% feeling ‘very confident’ in their ability to perform CVC insertion (Figure 4(b)).

## Discussion

To our knowledge, this is the first study describing outcomes using a longitudinal procedure simulation curriculum using low-fidelity, gelatin-based simulation models. We found that while intern US-guided CVC insertion competency did improve overall over the course of the year, there was still skill atrophy between testing sessions, as expected. While the single-group observational nature of our study precludes any conclusions regarding the magnitude of the effect that our model had on these outcomes, survey data from interns indicated that interns did find our model useful. This paired with the fact that nearly half (47.9%) of all interns indicated that they wanted more practice with ultrasound and a large majority (85.4%) did not want to practice on a higher fidelity model suggests that the use of low-cost, low-fidelity, gelatin-based models to simulate obtaining US-guided vascular access should be studied in larger, prospective, randomized controlled trials.

In a perfect world, trainees in IM would have portable, reusable, high-fidelity, low-cost US-guided CVC insertion models available to them anytime they needed to practice. In this ideal scenario, interns and residents would also have sufficient time to practice on their models immediately prior to attempting a procedure on a patient. In reality, simulation in medical education is limited by a litany of factors including cost and portability of available models [3–13,32,33]. While medical educators have overcome these problems to some degree with innovative new models, our study examined the utility of a novel low-cost, reusable, portable, and bacteria-resistant gelatin-based model that was available to all interns in an ICU throughout the course of their rotation. We specifically ensured that our models were available to interns 24 hours per day while on their rotation and reinforced the importance of practicing immediately prior to any procedures. Since the total cost of the materials needed to construct each model is roughly 6 USD (US), all are homemade, all are bacteria-resistant at room temperature, and each can be made within 3 hours, models were easily replaceable and interns and residents could easily request extra if necessary. In these ways, this model design provides a significant advantage over other models available in the literature and allows for the convenience of being available to interns at the point of care immediately prior to performing their procedure.

While our study was not designed to quantify the magnitude of the effect of a novel US-guided CVC insertion model on metrics of procedural competency, it did reveal several important observations for medical educators. First, data collected during the course of our study reflect findings of studies conducted in surgical literature that show that the frequency of performing operative procedures correlates with patient outcomes [34–36]. Interns included in our trial demonstrated a rapid loss of competency across several skills (time to flash, number of sticks, and needle tracking) between initial boot camp training and the start of their ICU month during their first year of training. After being immersed in an ICU environment for a month, which also included CVC insertion simulation on gelatin-based models, their skills rapidly improved but then waned throughout the year. While these results are somewhat intuitive and expected, this study validates the application of surgical data supporting the importance of frequent procedural practice in the maintenance of skill competency to interns in IM [14,15]. Our data can also help anticipate rates of competency acquisition, loss of procedural skills, and baseline intern experience levels in future studies.

Interestingly, despite the majority of interns describing the gelatin-based models as ‘somewhat helpful’ or ‘very helpful,’ model use was less than anticipated. It is unclear if this was due to model-based factors, such as lack of ongoing utility after initial use, or external factors, such as limited time or access to an US. This will need to be investigated in detail in further studies to elucidate the exact barriers to model use.

Our study had multiple limitations. First, we did not have a control group by design. Multiple well-designed, randomized studies from medical and surgical literature have demonstrated the benefits of simulation in medical education and therefore we chose to use a single experimental group as we believed this would give us useful information that could inform future studies without compromising the education of our residents [22–25]. Next, we were unable to measure the impact of model use on complications of clinical procedures, which limited our ability to directly correlate procedure simulation and practice with patient safety outcomes. Finally, the entire CVC insertion procedure was not evaluated, as our models were specifically designed to simulate just the US-guided needle insertion portion of the procedure. Therefore, we were not able to measure outcomes such as instances of deviation from sterile technique, total time to completion of procedure, or deviation from standard procedure protocols.

In conclusion, we designed a longitudinal simulation training curriculum using a point-of-care, portable, reusable, low-cost gelatin-based model designed to simulate US-guided CVC insertion. The results of our single group, prospective, observational trial are consistent with findings in other studies and do suggest a role for this type of model in medical education, but more research is needed to determine the optimal amount and timing of simulation training.

## Supplementary Material

Supplemental Material
