# Supplementary material for: Longitudinal trends using a point-of-care gelatin-based model for ultrasound-guided central venous catheter insertion
Source: Med Educ Online. 2021 May 7;26(1):1924350. doi: 10.1080/10872981.2021.1924350 (PMC8118402; doi:10.1080/10872981.2021.1924350)
Supplement: Supplemental Material [file ZMEO_A_1924350_SM2163.docx]

**Supplementary Data**

**Supplementary Table 1. Prototype Model Survey Responses***

| Characteristic | Responses |
| --- | --- |
| Type of Provider – no. (%) |  |
| Attending Physician  Fellow  Advanced Practice Provider | 2 (10.5)  4 (21.1)  13 (68.4) |
|  |  |
| Mean qualities of gelatin-based model – mean±SD (range) |  |
| Ultrasound appearance  Feel  Utility for teaching novice providers  Utility for teaching needle tracking  Overall utility | 2.6±0.5 (2-3)  2.6±0.5 (2-3)  2.6±0.5 (2-3)  2.7±0.5 (2-3)  2.8±0.4 (2-3) |
|  |  |

* Percentages may not total 100 because of rounding.

**Supplementary Table 2. Venous Access Competency Data***

| Parameter | Interns that completed all CVC testing  (n=40) |
| --- | --- |
| Median Time to Flash (IQR) |  |
| Post-Boot Camp  Pre-ICU  Post-ICU  End of Year | 34 (17.25-53)  37 (22.5-122.3)  15.5 (12.25-23)  22.5 (15.75-36.75) |
| Median Number of Sticks (IQR, 95% CI) |  |
| Post-Boot Camp  Pre-ICU  Post-ICU  End of Year | 1 (1-1, 1.1-1.5)  1 (1-2.75, 1.4-2.5)  1 (1-1, 1-1.1)  1 (1-1, 1-1.3) |
| Adequately Tracking Needle – no. (%) |  |
| Post-Boot Camp  Pre-ICU  Post-ICU  End of Year | 25 (62.5)  23 (57.5)  34 (85)  31 (77.5) |
| Recognition of Artery and Vein – no. (%) |  |
| Post-Boot Camp  Pre-ICU  Post-ICU  End of Year | 35 (87.5)  38 (95)  40 (100)  39 (97.5) |

* Percentages may not total 100 because of rounding.

**Supplementary Figure 1 – US-guided CVC insertion educational curriculum design**

**Model Creation**

1. Create models based on instructions (*How to make vascular model*).

2. Models will be made in 4 varieties.

i. Artery medial and inferior to vein, not tangential

ii. Artery medial and inferior to vein, tangential

iii. Artery medial and inferior to a vein with tortuous course, tangential

iv. Artery directly superior to vein, tangential

3. A new set of models (i-iv) will be used for each training session (i.e. per 8-12 residents in each boot camp session and each beginning of the month in the ICU)

i. Boot camp total: 4 x 1 boot camp (4)

ii. Beginning of the month total: 4 x 12 months (48)

4. Two new model ii’s will be used for each testing session

i. Boot camp: each half day 2 x 2 (4)

ii. Beginning of the month: 12 x 2 (24)

iii. End of the month: 12 x 2 (24)

iv. End of the year: 6 sessions x 2 (12)

**Boot Camp Design**

Phase 0: validation (June 2018)

1. Sample of senior residents, critical care fellow, critical care nurse practitioners/physician assistants and attending physicians will be provided models to assess and Blue Phantoms to compare

2. Survey (*Vascular access validity survey*) will be given after provider assess model and Blue Phantom

Phase 1: Boot camp training session (June 27, 2018)

1. Introduction structure script: 5 minutes (*Attached Introduction Script*)

2. Ultrasound guided vascular access training: 25 minutes

a. 2-3 interns work with one supervising physician (fellow or attending)

b. Supervising physician first demonstrates how to identify artery and vein on model

i. Lateral

ii. Superior

iii. Compressible

c. Supervising physician demonstrates how to track needle tip

d. Intern practices on model with assistance and coaching from supervising physician with remaining time

3. Post course assessment (immediately after vs end of day vs another day during boot camp: To be determined by IM program directors)

a. One intern to one supervisor at a time.

i. Model, needle, syringe, ultrasound and gel available.

b. Time Session structured script is read (*Attached Time Session Script*)

c. Supervising physician will begin timing at end of script and record measures

i. Appropriately identifies artery (lateral, superior, compressible)

ii. Number of attempts (# with attempt counting as needle in the silicon cover)

iii. Time to blue flash (min:sec)

iv. Needle tip tracked (Y/N)

v. Needle tip visualized in vessel (Y/N)

4. Survey to be given to intern at end of testing. (*attached vascular access training survey*)

Phase 2: Beginning of Grady MICU month training session (3-5th day of each calendar month from July 2018-June 2019: Time and dates to be determined by MICU directors)

1. Introduction structure script: 5 minutes (*Attached Introduction Script*)

2. Ultrasound guided vascular access training: 25 minutes a. 2-3 interns work with one supervising physician (fellow or attending)

b. Supervising physician first demonstrates how to identify artery and vein on model

i. Lateral

ii. Superior

iii. Compressible

c. Supervising physician demonstrates how to track needle tip

d. Intern practices on model with assistance and coaching from supervising physician with remaining time

3. Post course assessment (immediately after session)

a. One intern to one supervisor at a time.

i. Model, needle, syringe, ultrasound and gel available.

b. Time Session structured script is read (*Attached Time Session Script*)

c. Supervising physician will begin timing at end of script and record measures

i. Appropriately identifies artery (lateral, superior, compressible)

ii. Number of attempts (# with attempt counting as needle in the silicon cover)

iii. Time to blue flash (min:sec)

iv. Needle tip tracked (Y/N)

v. Needle tip visualized in vessel (Y/N)

4. Survey to be given to intern at end of testing (*attached vascular access training survey*)

Phase 3: Continued self-education

1. During Grady MICU month, interns are expected to practice stick to flash 5 x per week

2. Fellow and attendings may assist in education during these sessions

3. Residents will be on honor system to report this

Phase 4: End of Grady MICU month testing session (27-31st day of each calendar month from July 2018-June 2019: Time and dates to be determined by MICU directors)

1. Post course assessment (immediately after session)

a. One intern to one supervisor at a time.

i. Model, needle, syringe, ultrasound and gel available.

b. Time Session structured script is read (*Attached Time Session Script*)

c. Supervising physician will begin timing at end of script and record measures

i. Appropriately identifies artery (lateral, superior, compressible)

ii. Number of attempts (# with attempt counting as needle in the silicon cover)

iii. Time to blue flash (min:sec)

iv. Needle tip tracked (Y/N)

v. Needle tip visualized in vessel (Y/N)

2. Survey to be given to intern at end of testing. Including question on how many practice sessions and attempts. (*attached vascular access end of Grady training survey*)

Phase 5: End of year session during resident responsiveness testing session (April-June 2018)

1. Done at end of course, 1630-1700 time slot

a. One intern to one supervisor at a time.

i. Model, needle, syringe, ultrasound and gel available.

b. Script is read:

i. Intern asked to identify vein and state how they are identifying vein (lateral, superior, compressible)

ii. Intern asked to aspirate venous blood

iii. Intern asked to vocalize and demonstrate when needle tip is in vein

c. Supervising physician will begin timing at end of script and record measures

i. Appropriately identifies artery (lateral, superior, compressible)

ii. Number of attempts (# with attempt counting as needle in the silicon cover)

iii. Time to blue flash (min:sec)

iv. Needle tip tracked (Y/N)

v. Needle tip visualized in vessel (Y/N)

2. Survey to be given to intern at end of testing (*attached vascular access training survey*)

Introduction Script (5 minutes)

Today you are going to learn how to place an emergent ultrasound guided right internal jugular vein central line via this gelatin model. We will first demonstrate how to identify the vein with the ultrasound. Next we will demonstrate how to actively track the needle tip using the ultrasound. You will then have 25 minutes to practice using the supplied equipment, including the ultrasound machine. At the end of the session we will ask you to:

1. Identify the vein and then verbalize your criteria for identification to the supervisor

2. Access the vein using the supplied needle and then verbalize when needle tip has entered the vessel

Time Session Script (5 minutes)

We’d like you to now proceed with placing an emergent ultrasound guided right internal jugular vein central line via this gelatin model. We’d ask that you operate under the assumption that consent has been obtained and that you are performing this under the appropriate sterile precautions. We would like for you to use the supplied equipment, including the ultrasound machine. We will ask you to:

1. Identify the vein and then verbalize your criteria for identification to the supervisor

2. Access the vein using the supplied needle and then verbalize when needle tip has entered the vessel

Vascular Access Time Session Measurements

1. Resident appropriately identifies artery? (Check all that apply)

____Lateral ____Superior ____Compressible

2. Number of attempts (each time needle punctures silicone is an attempt)? ______________

3. Time to flash (when blue is free flowing in syringe)? ______ mins ______ seconds

4. Was needle tip tracked while accessing vessel? ______ Yes _______ No

5. Was needle tip visualized in vessel at the time resident achieved flash?

______ Yes ______ No

**Supplementary Figure 2 – Surveys**

Vascular Access Validity Survey

1. What is your current position? (Please check one – if PGY, please include current year of training)

____PA ____NP ____Attending MD ____PGY ____Other

2. Over the past year, approximately how many ultrasound guided central venous lines have you placed? _______

For the next series of questions, please provide an answer based on the following rating system:

1 – Inferior to other vascular models you have previously trained on

2 - Comparable to other vascular models you have previously trained on

3 - Superior to other vascular models you have previously trained on

3. How realistic would you rate the appearance on ultrasound of the gelatin vascular model that you used today? _____

4. How realistic would you rate the feel of obtaining vascular access on the gelatin vascular model as compared to a live patient? _____

5. How effective do you think the gelatin vascular model is in teaching novice providers to distinguish between an artery and vein? _____

6. How effective do you think the gelatin vascular model is in teaching novice providers to track their needle when obtaining vascular access? _____

7. Overall, how effective do you think the gelatin vascular model is in teaching novice providers the technical skill needed to successfully cannulate a central vein? _____

Vascular Access Post-Boot Camp Survey

1. What is your current position? (Please check year and training)

____PGY1 _____Categorical

____PGY2 _____Preliminary

____PGY3 _____Other

____PGY4

2. Over the past year, approximately how many ultrasound-guided central venous lines have you placed? ______________

3. Over the past year, approximately how many ultrasound-guided peripheral IVs have you placed? _____________

For the next series of questions, please provide an answer based on the following rating system:

1 – Not at all

2 – Moderately confident

3 – Very confident

4. How comfortable would you feel performing a central line placement unsupervised?

5. How comfortable would you feel performing an arterial line unsupervised?

6. How confident are you in your ability to successfully place a central line alone?

7. How confident are you in your ability to successfully place an arterial line alone?

8. How helpful did you find this course? (Circle one)

Not at all

Somewhat helpful

Very helpful

Vascular Access Post-ICU Survey

1. What is your current position? (Please check year and training)

____PGY1 _____Categorical

____PGY2 _____Preliminary

____PGY3 _____Other

____PGY4

For the next series of questions, please provide an answer based on the following rating system:

1 – Not at all

2 – Moderately confident

3 – Very confident

4. How comfortable would you feel performing a central line placement unsupervised?

5. How comfortable would you feel performing an arterial line unsupervised?

6. How confident are you in your ability to successfully place a central line alone?

7. How confident are you in your ability to successfully place an arterial line alone?

8. How many times did you practice on gelatin models during the month?

Less than once weekly

1-5 times weekly

More than 5 times weekly

Vascular Access End of Year Training Survey

1. What is your current position? (Please check year and training)

____PGY1 _____Categorical

____PGY2 _____Preliminary

____PGY3 _____Other

____PGY4

2. Over the past year, approximately how many ultrasound guided central venous lines have you placed? ______________

3. Over the past year, approximately how many ultrasound guided arterial lines have you placed? _____________

For the next series of questions, please provide an answer based on the following rating system:

1 – Not at all

2 – Moderately confident

3 – Very confident

4. How comfortable would you feel performing a central line placement unsupervised? _____

5. How comfortable would you feel performing an arterial line unsupervised? _____

6. How confident are you in your ability to successfully place a central line alone? _____

7. How confident are you in your ability to successfully place an arterial line alone? _____

8. How confident are you in supervising a central line placement? _____
